# Increased Levels of S100A8/A9 in Patients with Peritonsillar Abscess: A New Promising Diagnostic Marker to Differentiate between Peritonsillar Abscess and Peritonsillitis

**DOI:** 10.1155/2017/9126560

**Published:** 2017-10-17

**Authors:** Christoph Spiekermann, Antonella Russo, Markus Stenner, Claudia Rudack, Johannes Roth, Thomas Vogl

**Affiliations:** ^1^Department of Otorhinolaryngology, Head and Neck Surgery, University Hospital Münster, Münster, Germany; ^2^Institute of Immunology, University Hospital Münster, Münster, Germany

## Abstract

Peritonsillar abscess (PTA) is a very frequent reason for urgent outpatient consultation and otolaryngological hospital admission. Early, correct diagnosis and therapy of peritonsillar abscess are important to prevent possible life-threatening complications. Based on physical examinations, a reliable differentiation between peritonsillar cellulitis and peritonsillar abscess is restricted. A heterodimeric complex called calprotectin consists of the S100 proteins A8 and A9 (S100A8/A9) and is predominantly expressed not only in monocytes and neutrophils but also in epithelial cells. Due to its release by activated phagocytes at local sites of inflammation, we assumed S100A8/A9 to be a potential biomarker for peritonsillar abscess. We examined serum and saliva of patients with peritonsillitis, acute tonsillitis, peritonsillar abscess, and healthy controls and found significantly increased levels of S100A8/A9 in patients with PTA. Furthermore, we could identify halitosis, trismus, uvula edema, and unilateral swelling of the arched palate to be characteristic symptoms for PTA. Using a combination of these characteristic symptoms and S100A8/A9 levels, we developed a PTA score as an objective and appropriate tool to differentiate between peritonsillitis and peritonsillar abscess with a sensitivity of 92% and specificity of 93%.

## 1. Introduction

The palatine tonsils are related to the mucosa-associated lymphatic tissue (MALT) of the upper respiratory tract. Immunologically, four important microcompartments can be defined: the crypt epithelium which consists of a nonuniform epithelium; the follicular germinal center; the mantle zone, which is characterized by a high density of lymphocytes; and the interfollicular area, populated by T-lymphocytes [1]. As a part of Waldeyer's ring, tonsils play an important role in nasopharyngeal immunity and present the first line in host defense against pathogens particularly in children. In some cases, however, they trigger severe head and neck infections causing life-threatening complications [2, 3]. The most common severe head and neck infection is the peritonsillar abscess (PTA) which is a very frequent reason for nonelective otolaryngological hospital admission [4]. Extension of an acute tonsillitis or an infection of Weber's salivary glands in the supratonsillar fossa was discussed to be the associated pathomechanisms of PTA which is characterized by an accumulation of pus between the fibrous capsule of the palatine tonsil and the pharyngeal constrictor muscle [5, 6]. Due to the level of the uvula tip, peritonsillar abscesses can be divided into superior and inferior types. With a percentage of 75%, the superior type is described to be the more common sort of peritonsillar abscesses [3].

Bacteria could be detected in abscess aspirates in 98% of the cases and 76% of those cases showed a combination of anaerobic and aerobic isolates [7, 8]. *Fusobacterium necrophorum* and *Prevotella* were described to be the most frequent anaerobic bacteria, and *Streptococcus pyogenes*, *Staphylococcus aureus*, and *Haemophilus influenzae* were the common aerobic isolates from peritonsillar abscess [7–11].

Incisional drainage, needle aspiration, and tonsillectomy are common treatment strategies in peritonsillar abscesses. Indications of abscess tonsillectomy became more doubtful over the years because this procedure is associated with an increased risk of spread of infection and postoperative hemorrhage [3]. Thus, needle aspiration was established as a less invasive, alternative treatment approach for peritonsillar abscess with success rates between 72 and 95% [12–14]. Recurrence of a peritonsillar abscess could be observed in 10–19% after needle aspiration and the need of multiple attempts for adequate abscess relief indicate the strains for the patients and the limitation of this therapeutic regime [12, 15]. Incision and drainage of the peritonsillar abscess is considered to be the more definite but also more painful procedure [16]. Only medical therapy has been described to be as successful as initial surgical treatment in patients with less severe initial infection [4]. Due to these highly variable and partially expensive therapeutic regimes, the optimal treatment strategy of patients with PTA at reasonable cost level still remains controversial [12, 16].

Furthermore, there is a small group of patients who show symptoms suspicious of PTA such as trismus, uvula edema, and swelling of the arched palate. Still, aspiration or incisional drainage revealed no pus. In this group, diagnosis changed from PTA to peritonsillar cellulitis (PC), also known as peritonsillitis [17]. Reliable differentiation between PTA and PC is of great importance to avoid delay of the appropriate treatment and consequently life-threatening complications like airway obstruction, aspiration and pneumonia, or erosion of major vessels [9, 18]. However, 50% of the PTA patients are treated by nonotolaryngologists and diagnosis based on clinical examination is associated with a sensitivity of 78% and a specificity of just 50% [13, 18]. Hence, objective criteria or biomarkers to identify patients with PTA and to discriminate PTA from PC, to assess the severity of infection, or to identify patients who benefit from medical or surgical treatment would be helpful but still remain desirable [14].

S100A8 and S100A9, also known as myeloid-related proteins 8 and 14 (MRP 8/14), are members of the S100-protein family and show proinflammatory activities in a variety of different diseases. Both proteins form heterodimers also known as calprotectin or tetramers in the presence of calcium ions and are not only predominantly expressed in monocytes and neutrophilic granulocytes but are also inducible in epithelial cells [19, 20]. They are related to the group of danger-associated molecular patterns (DAMP) or alarmins and activate leukocytes via a toll-like receptor 4 (TLR4) pathway resulting in increased cytokine and chemokine expression and thereby trigger inflammatory reactions [21–23]. There have been several reports about the pivotal role of S100A8/A9 as a biomarker in inflammatory diseases like rheumatoid arthritis, acute myocardial infarction, or chronical inflammatory bowel diseases [24–26].

In this prospective study, we examined S100A8/A9 levels in the serum and saliva and its potential role as a promising and helpful biomarker to differentiate between acute tonsillitis (AT), PC, and PTA.

## 2. Material and Methods

### 2.1. Patients and Healthy Controls

This prospective study was performed in the Department of Otorhinolaryngology, Head and Neck Surgery, University Hospital Münster. 25 patients with acute tonsillitis (AT) (11 males, 14 females, 27.8 ± 2.5 years [mean ± SEM]), 36 patients suffering from peritonsillar abscess (19 males, 17 females, 34.4 ± 2.8 years), and 16 patients with peritonsillitis (8 males, 8 females, 34.8 ± 4.8 years) were included. Peritonsillar abscess was diagnosed by needle aspiration, abscess drainage in local or tonsillectomy in common anesthesia. Patients with clinical presentation similar to PTA but negative needle aspiration, incision, or tonsillectomy without revealing any purulent fluid and a good response to systemic antimicrobial treatment were assigned to the peritonsillitis group. Diagnosis was proven retrospectively. Healthy volunteers (*n* = 15, 32.9 ± 10.5 years) without any history of acute or recurrent tonsillitis served as controls. Symptoms and observations of the physical examination were documented during initial outpatient consultation. The study was approved by the institutional ethics committee [2015-217-f-S], and written informed consent was obtained from all subjects.

### 2.2. S100A8/A9 Sandwich ELISA of the Serum and Saliva Samples

Serum samples were centrifuged at 2000*g* for 10 minutes within 2 hours after acquisition, and supernatant was aliquoted and stored at −20°C until analysis. Saliva acquisition was performed with untreated Salivette® (Sarstedt, 51.1534) as described in the manufacturer's datasheet or by collecting saliva in a 50 ml Falcon tube and centrifugation at 1000*g* for 15 minutes. Supernatant was aliquoted and stored at −20°C. S100A8/A9 concentrations were measured with a sandwich enzyme-linked immunosorbent assay (ELISA) for human S100A8/A9 as described earlier [27].

### 2.3. Bead-Based Immunoassay

Quantification of cytokines/chemokines in serum and saliva was performed with the LEGENDplex™ assay “Human Inflammation Panel” (BioLegend) as described in the manufacturer's manual. The “Human Inflammation Panel” allows simultaneous measurement of IL-1*β*, IL8, and other cytokines and chemokines. Fluorescent signal intensities were detected by NAVIOS™ Flow Cytometer (Beckmann Coulter).

### 2.4. Laboratory Parameters

As common inflammatory parameters, C reactive protein (CRP, [mg/dl]) and whole white blood cells [×10^3^/*μ*l] were analyzed by clinical routine methodology.

### 2.5. Histological and Immunohistochemical (IHC) Analyses

Tonsils of 10 patients undergoing tonsillectomy because of peritonsillar abscess were used for further histological examinations. As a healthy control, hypertrophic tonsils of patients without any history of recurrent tonsillitis were obtained (*n* = 10). The tonsils were divided into two parts immediately after surgical extirpation. One-half was snap-frozen in liquid nitrogen and stored at −80°C, whereas the other part was fixed in 4% formalin for 2-3 days. Samples were embedded in paraffin and cut into 3 *μ*m thick sections followed by staining with hematoxylin and eosin. Furthermore, polyclonal rabbit anti-human S100A8 and anti-human S100A9 were used as primary antibodies and a biotinylated goat anti-rabbit IgG was added as a secondary antibody. Streptavidin peroxidase binding to biotin and its reaction with 3′-amino-ethyl-carbazole (Sigma, Germany) was utilized to identify S100A8/A9 localization in the tonsils. Additionally, nuclear counterstaining was performed with Mayer's hämalaun (Merck, Germany). Microphotographs of the complete tonsils were performed with the AxioVision MosaiX module for the Axio Observer Z1 microscope (Zeiss, Germany). Using a four-point Likert scale (0 = no staining, 1 = mild staining, 2 = moderate staining, and 3 = high positive staining), semiquantitative analysis of the stained sections was performed by two independent investigators who were blinded regarding the diagnosis.

### 2.6. Statistical Analysis

Results are mean values ± standard error of the mean (mean ± SEM) or mean value ± standard deviation (mean ± SD) as indicated in the figures. Chi-square analysis was used to identify possible relations between variables. Student *t*-test was used to detect significant differences in parametric results and Mann–Whitney *U* test was performed to analyze differences between nonparametric groups. *p* values > 0.05 are considered not to be significant. Significant results are marked with asterisks (^∗^*p* < 0.05, ^∗∗^*p* < 0.01, and ^∗∗∗^*p* < 0.001). The capacity of the model to differentiate between positive and negative results is illustrated by receiver operating characteristic (ROC) curves which allow calculation of area under the curve values (*A* values) and cut-off values. Discriminative power of the model is considered to be excellent with an *A* value of >0.9, good > 0.8, acceptable > 0.7, and poor > 0.6. Statistical analyses and creation of figures were performed with IBM® SPSS® Statistics 24 and SigmaPlot®12.

## 3. Results

### 3.1. Serum and Saliva Analysis

**Figure 1:**
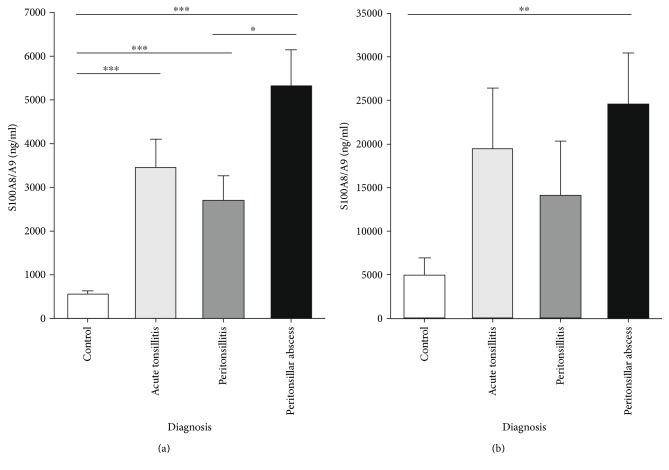
S100A8/A9 levels in serum (a) and saliva (b) of controls and patients with acute tonsillitis, peritonsillitis, or peritonsillar abscess (mean ± SEM). All entities show significantly increased serum levels compared to the controls. Furthermore, significantly higher S100A8/A9 levels were detectable in sera of PTA patients compared to PC patients. There was a significant increase of salivary S100A8/A9 levels in patients with PTA compared to healthy controls (^∗^*p* < 0.05, ^∗∗^*p* < 0.01, and ^∗∗∗^*p* < 0.001).

**Figure 2:**
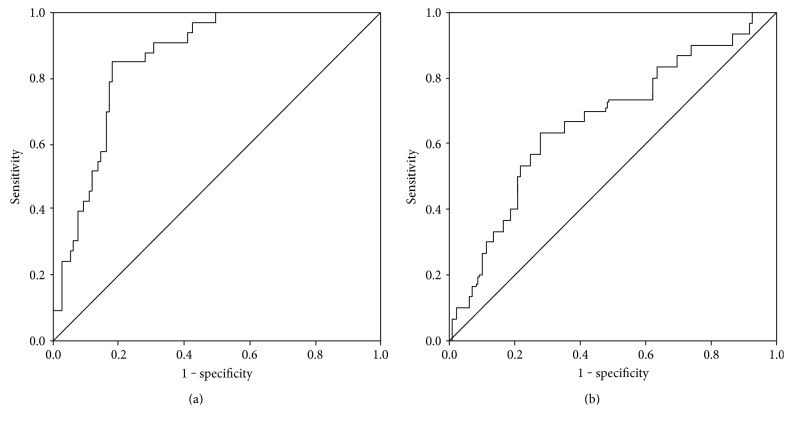
Receiver operating characteristic (ROC) curve in black, sensitivity on *y*-axis, and 1 − specificity on *x*-axis of S100A8/A9 in serum (a) and saliva (b) to identify patients with peritonsillar abscess. Grey: diagonal association line. Area under the curve (a) = 0.86 and (b) = 0.67.

**Figure 3:**
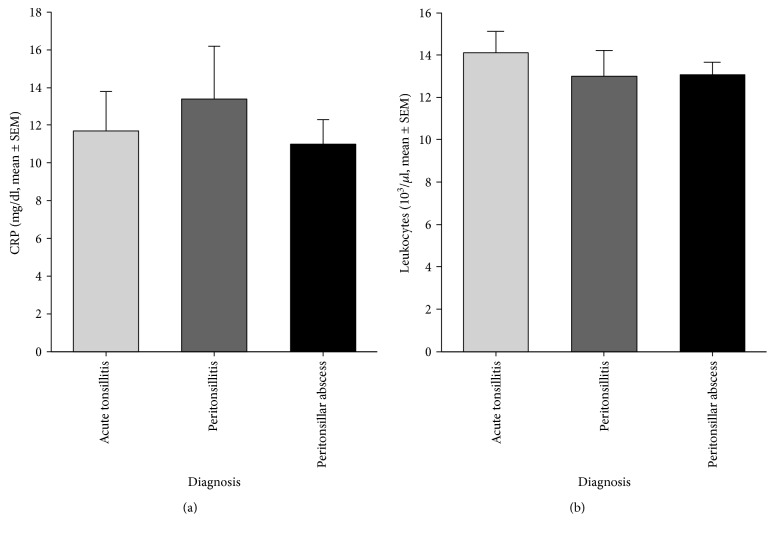
CRP levels (a) and leukocytes (b) in patients with acute tonsillitis, peritonsillitis, and peritonsillar abscess (mean ± SEM). No significant differences were observed between cohorts.

**Figure 4:**
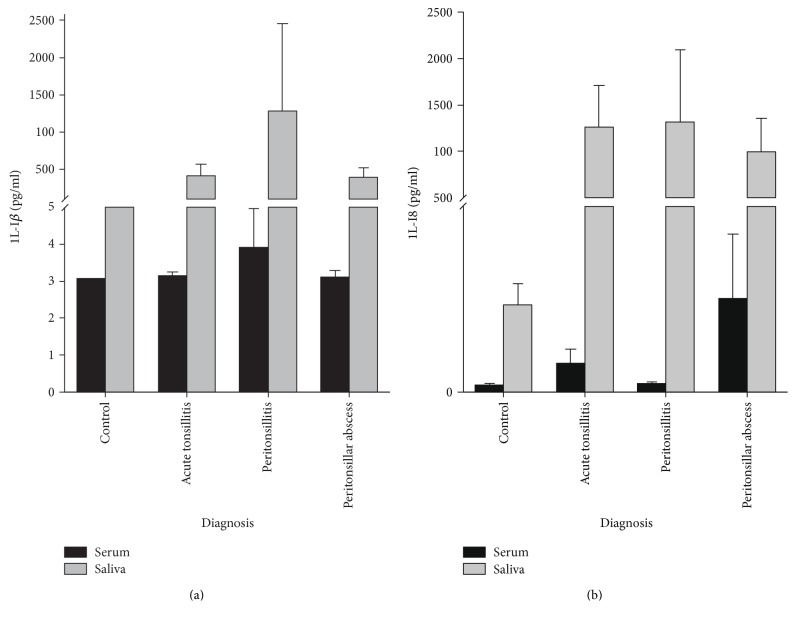
Cytokines and chemokines in saliva and serum. Levels of IL-1*β* (a) and IL-8 (b) in controls, patients with acute tonsillitis, peritonsillitis, or peritonsillar abscess. No significant differences between the diagnosis groups were detectable (*p* > 0.05). Black bars = serum levels, grey bars = saliva levels, mean ± SEM.

Systemic S100A8/A9 levels were increased in the serum of patients with acute tonsillitis compared to healthy controls (3450 ± 650 ng/ml versus 550 ± 90 ng/ml, *p* < 0.001). Furthermore, S100A8/A9 levels in patients with PTA (5330 ± 820 ng/ml) were significantly higher than in patients with PC (2710 ± 550 ng/ml, *p* < 0.05) and healthy controls (*p* < 0.001). There was no significant difference in S100A8/A9 levels in the sera between patients suffering from AT and PC or PTA (Figure 1(a)). Analysis of S100A8/A9 levels in saliva revealed no significant difference of the S100A8/A9 level in patients with AT (19480 ± 6920 ng/ml, *p* = 0.08) or PC (14110 ± 6220 ng/ml, *p* = 0.265) in comparison to the control group (4940 ± 1980 ng/ml). However, the S100A8/A9 level in patients' saliva with peritonsillar abscess was significantly higher than in controls (24590 ± 5850 ng/ml, *p* = 0.002) (Figure 1(b)). Although a difference between the PTA and the PC group could be observed, the results were not significant (*p* = 0.087). Receiver operating characteristic (ROC) curve analysis provided a cut-off value of 8180 ng/ml in saliva (sensitivity = 0.63, specificity = 0.72, *p* = 0.019) and 2550 ng/ml in serum (sensitivity = 0.85, specificity = 0.82, *p* = 0.001) for the existence of a peritonsillar abscess (Figures 2(a) and 2(b)). Neither levels of CRP (AT: 9.82 ± 1.82 mg/dl, PTA: 11.99 ± 1.45 mg/dl, and PC: 12.69 ± 2.43 mg/dl, *p* > 0.05) nor levels of white blood cells (AT: 13.38 ± 0.90 × 10^3^/*μ*l, PTA: 14.11 ± 0.76 × 10^3^/*μ*l, and PC: 13.08 ± 1.15 × 10^3^/*μ*l, *p* > 0.05) showed any significant differences between the diagnosis groups (Figures 3(a) and 3(b)). Also, analysis of IL-1*β* and IL-8 in sera and saliva revealed no significant differences between the different cohorts (Figure 4).

### 3.2. Histological and Immunohistochemical Examinations

**Figure 5:**
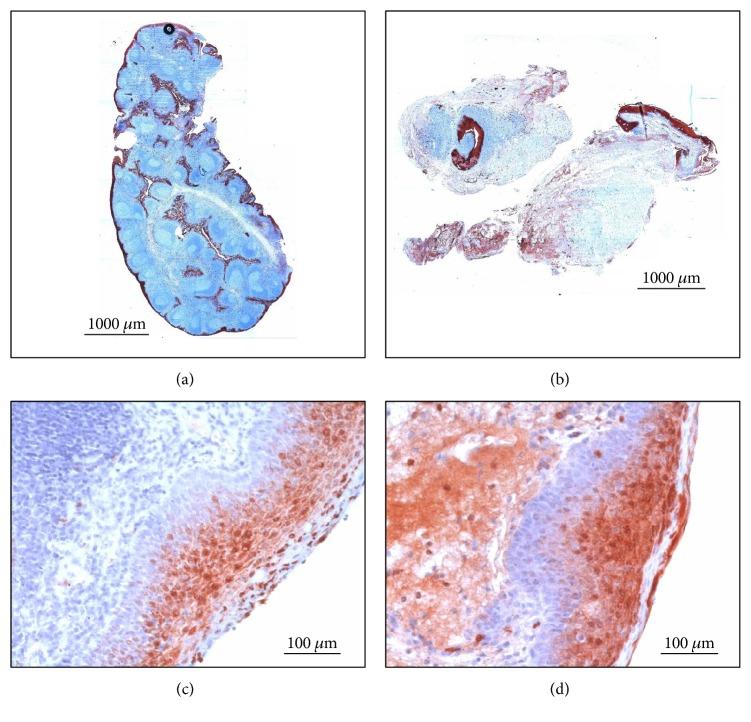
IHC staining for S100A8 of human palatine tonsils of patients with hypertrophic tonsils without any history of recurrent tonsillitis (a, c) and suffering from peritonsillar abscess (b, d). S100A8 expression is limited to the surface and crypt epithelium of the hypertrophic tonsil. The tonsil of the PTA patient shows an intensified staining for S100A8 in the epithelium and the parenchyma. (a) and (b): MosaiX module, original magnification ×40. (c) and (d): magnification ×200.

PTA is a localized process with an accompanying inflammatory reaction. Hence, to avoid misinterpretation of sectional S100A8/A9 expression, the MosaiX module was utilized to analyze each tonsil in toto. A normal microarchitecture with crypt epithelium, follicular germinal centers was observed in the tonsil from patients with PTA. Staining for S100A8/A9 was strongly positive in tonsils of patients with PTA (3.30 ± 0.23 [mean score ± SD]) in contrast to hyperplastic tonsils without any history of tonsillitis (1.68 ± 0.22, *p* < 0.001). Above all, there was a sectional positive staining which might be due to abscess localization (Figures 5(a), 5(b), 5(c), and 5(d)).

### 3.3. Development of a PTA Score

**Figure 6:**
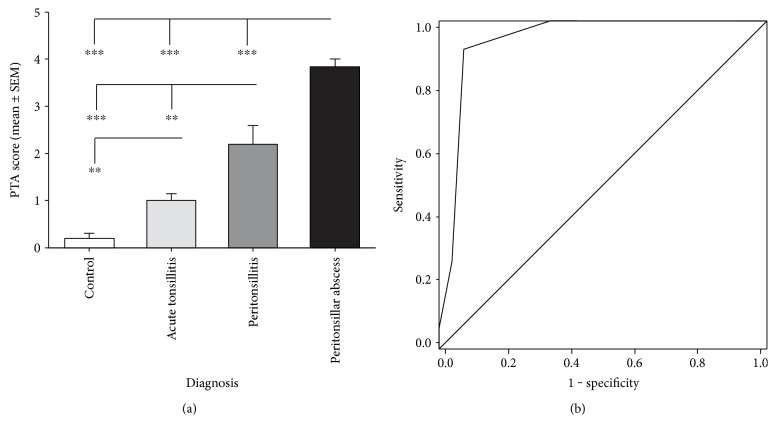
PTA score (*S*_PTA_) was developed by the addition of one point each for symptoms like trismus, halitosis, uvula edema, unilateral swelling of the arched palate, S100A8/A9 > 2550 ng/ml in the serum, and S100A8/A9 > 8180 ng/ml in the saliva. Patients with acute tonsillitis, peritonsillitis, and peritonsillar abscess show significant higher *S*_PTA_ values in comparison with the controls. Furthermore, a significant difference of *S*_PTA_ values between the PTA and the PC cohort was detectable (a). ROC analysis of the PTA score revealed a cut-off value of *S*_PTA_ = 2.5 (sensitivity = 0.92, specificity = 0.93, *p* < 0.001) to identify patients suffering from PTA (black: ROC curve in black, grey: diagonal association line) (^∗∗^*p* < 0.01, and ^∗∗∗^*p* < 0.001).

Diagnosis of PTA is made by clinical examination and depends on subjective assessment of the clinician. Chi-square analysis revealed no significant relations between CRP (*χ*^2^ = 124.05, *p* = 0.431), leukocyte levels (*χ*^2^ = 135.8, *p* = 0.301), and patients with peritonsillar abscess. Symptoms like trismus (*χ*^2^ = 30.39, *p* < 0.001), halitosis (*χ*^2^ = 12.14, *p* = 0.007), uvula edema (*χ*^2^ = 27.01, *p* < 0.001), and unilateral swelling of the arched palate (*χ*^2^ = 60.11, *p* < 0.001) were observed to be helpful clinical characteristics to identify peritonsillar abscess. Hence, by addition of one point for each symptom and for S100A8/A9 levels above the cut-off value of 2550 ng/ml in serum or 8180 ng/ml in saliva, a PTA score (*S*_PTA_) with the lowest value of 0 and a maximum value of 6 points was developed (Table 1). PTA (*S*_PTA_ = 3.84 ± 0.15, *p* < 0.001), PC (*S*_PTA_ = 2.13 ± 0.38, *p* < 0.001), and AT (*S*_PTA_ = 0.93 ± 0.22, *p* = 0.002) showed significantly increased *S*_PTA_ values in contrast to the control group (*S*_PTA_ = 0.2 ± 0.11). Furthermore, the differences between AT and PTA or PC were significant (PTA: *p* < 0.001, PC: *p* = 0.007) as well as the difference between patients suffering from PTA and PC (*p* < 0.001) (Figure 6(a)). A ROC curve analysis revealed a cut-off value of 2.5 (sensitivity = 0.92, specificity = 0.93, *p* < 0.001) for the existence of a peritonsillar abscess (Figure 6(b)). The likelihood of PTA increases with higher *S*_PTA_ values (Table 2). For *S*_PTA_ values ≥ 3, the overall probability of PTA is about 89.2%.

## 4. Discussion

Several studies analyzed the influence of lymphocytes and the adaptive immune system on acute and recurrent tonsillitis and the triggering pathogens [2, 5, 11]. To our knowledge, this is the first report of a correlation between elevated expression of S100A8/A9 and AT, PC, and PTA suggesting an important role of the innate immune response in disease development. As already mentioned above, S100A8/A9 has been described as a biomarker in several inflammatory and malignant diseases [24, 28–31]. In this study, we could quantify increased S100A8/A9 levels in patients' sera and saliva suffering from PTA.

Although increased levels of CRP (>15.5 mg/dl) and age > 35 years are described to be predictors of retropharyngeal abscesses and necrotizing fasciitis, it is proven, and our data confirm, that neither CRP nor leukocytes in sera are appropriate markers to distinguish between PTA and AT [10]. Additionally, there are also no useful markers to differentiate between PTA and PC. Nevertheless, low correlations of S100A8/A9 levels in serum with CRP and leukocytes were detectable in patients with tonsillitis which is in concordance with recent findings in patients with myocardial infarction or rheumatoid arthritis [32, 33]. Recently increased levels of IL-8 in the tissue of tonsils with PTA compared to tonsils derived from patients with recurrent tonsillitis were observed, and strong positive correlations of S100A8/A9 and IL-8 have been described in association with congestive heart failure [34, 35]. Although we could observe a correlation between S100A8/A9 and IL-8 levels in the serum and saliva as well, neither IL-8 nor any other cytokine or chemokine we determined in the serum and saliva provides the potential to differentiate between acute tonsillitis, peritonsillitis, and peritonsillar abscess. Thus, we can assume S100A8/A9 to be a useful biomarker to identify patients with PTA.

Saliva of both patients and controls are macroscopically very inhomogeneous, and consequently, the content of S100A8/A9 shows a great variety in all cohorts. Although the data analysis has a high standard deviation, a comparative analysis was possible. Immunohistochemical findings revealed higher concentrations of S100A8/A9 in the tonsils of patients with PTA in contrast to hyperplastic tonsils without any history of tonsillitis. These findings impressively demonstrate a pivotal role in the pathomechanism as well as local expression and release of these DAMPs during PTA. However, the influence of S100A8/A9 on the development of PTA and its function regarding the tonsillar epithelium have not been elucidated so far. Hypothetically, S100A8/A9 is expressed by tonsil epithelial cells due to disruption of barrier function. A positive feedback mechanism with amplification of inflammation, induction of proinflammatory cytokine production, and simultaneous proliferation of keratinocytes as described in patients with systemic lupus erythematosus or psoriasis is assumable in tonsils and tonsillitis [36]. Thus, associated with leukocyte recruitment, pathogens could be eliminated and invasion could be averted [37]. Granulocyte migration through the gut wall into the feces allows determination of fecal S100A8/A9 in patients with inflammatory bowel diseases like Crohn's disease or appendicitis whereas release of S100A8/A9 by endothelial cells could be observed due to leukocyte interaction [26, 27, 37, 38]. According to these findings, two possible mechanisms for elevated salivary levels of S100A8/A9 could be assumed: (1) active secretion by the tonsillar keratinocytes and (2) migration of activated leukocytes through the tonsillar epithelium.

Patients with peritonsillitis show symptoms suspicious of peritonsillar abscess but early, correct, and reliable diagnosis is important to determine the adequate treatment approach and to prevent the progress of the PTA attended with severe complications. The difficulty to differentiate between PTA and PC in clinical examination points out the necessity of an objective, reliable parameter [14]. Computed tomography scan (CT) was considered to be a helpful tool to distinguish between PC and PTA [39]. CT scan might enhance the diagnostic accuracy and avoid unnecessary drainage procedures but there are some limitations which should be taken into consideration [40]. Grant et al. evaluated the CT scan in children with PTA and observed a delay of less-invasive interventions and no influence of the CT scan on the intervention chosen by the clinician [41]. Furthermore, in 13% of the 3 to 5-year-old children, common anesthesia or sedation was required for CT scan execution enhancing the risk of associated complications [41]. Due to the most important disadvantages of additional high costs, the radiation exposure and a specificity of just 50%, the value of the CT scan as an appropriate tool to differentiate between PTA and PC is doubtful. Thus, CT scan should be limited to special, isolated cases and not be part of routine PC or PTA management, particularly in children [41]. Hence, the intraoral and transcutaneous ultrasound of the tonsil and the peritonsillar abscess were established as a cost-efficient and lower-risk method compared to CT scan [18]. Intraoral ultrasound was described to be more sensitive than the transcutaneous ultrasound (89–95% versus 10–91%), and specificity of intraoral ultrasound varied from 70 to 83% [18, 42–45]. Although the ultrasound represents a noninvasive and rapid tool to differentiate between PTA and PC, there exist also some limitations restricting the use of this methodology. Albeit, transoral ultrasound was described to be well-tolerated; examination's result depends on the general cooperativeness as well as the physical condition of the patient [18]. Especially, children and compromised patients may not tolerate the procedure. Furthermore, in cases of severe trismus, entry of the probe into the oral cavity might be difficult or impossible [44]. Correct interpretation of the findings depends on the quality of ultrasound images as well as the advanced degree of technical and diagnostic expertise [18, 46]. Hence, further limitation arises due to limited access to trained ultrasonographers, a significant interuser variability, and limited availability of adequate equipment [18, 43, 44]. As mentioned above, 50% of the patients with PTA were treated by nonotolaryngologists who were probably not familiar with ultrasound of the head and neck region, and ultrasound is not available at every ambulance [13, 47]. Thus, for improving quality of care, it was the aim of the study to analyze the potential of S100A8/A9 as an objective marker to identify patients with peritonsillar abscess. We could show that the determination of S100A8/A9 levels in the serum and saliva in combination with symptoms suspicious of PTA is a helpful tool to distinguish between PTA and PC with a sensitivity of 92% and a specificity of 93%. Sample acquisition is not associated with any risks or complaints for the patients, and data analysis is independent from the degree of expertise. Hence, the newly developed PTA score seems to be an appropriate screening method for peritonsillar abscess. It should be mentioned that both the S100A8/A9 level in serum and saliva should be included in the new PTA score for improving sensitivity and specificity. If S100A8/A9 levels were excluded, a cut-off value of 0.5 for the existence of PTA was determined which is inadequate for differentiation between acute tonsillitis, peritonsillitis, and peritonsillar abscess. Analyzing the power of the PTA score without S100A8/A9 to differentiate PTA from PC and AT revealed a cut-off value of 1.5 with a sensitivity of 0.89 but a low specificity of 0.71. Thus, with a sensitivity of 92% and a specificity of 93%, the PTA score including S100A8/A9 levels in the serum and saliva is more reliable. Additionally, as mentioned above, increased levels of S100A8/A9 could be observed in serum of patients with varied diseases concerning the bowel, the joints, the heart, the skin, and multiple other organs, and enhanced S100A8/A9 levels have been observed in crevicular fluid of patients suffering from periodontitis [26, 27, 34, 48, 49]. Therefore, including typical symptoms of PTA, the PTA score indicates the probable association between elevated S100A8/A9 levels and PTA and is more reliable than the determination of S100A8/A9 levels solely.

However, this methodology also has its limitations. S100A8/A9 values were determined by ELISA which is time-consuming and not suitable for the outpatient consultation. Hence, a rapid test for the immediate and easy measurement of salivary and serum S100A8/A9 levels which can be performed by every person independent from degree of expertise, with a low interuser variability within a short time, is under development. Another limitation of our study is an incertitude concerning the diagnosis of peritonsillitis. Although needle aspiration or incision revealed no pus and patients showed a good response to intravenous antibiotics, an abscess could not be certainly excluded.

Further randomized clinical trials with bigger cohorts are necessary to verify the importance, sensitivity, specificity, and accuracy of the PTA score as a prognostic and diagnostic parameter in the future. Particularly, the possibility of the new developed *S*_PTA_ including S100A8/A9 to identify patients who might profit from medical treatment or whether tonsillectomy or abscess relief is required has to be elucidated. Furthermore, the influence of S100A8/A9 and the associated immune cells on abscess formation needs further investigations.

## 5. Conclusion

The elevated levels of S100A8/A9 in the sera and saliva of patients with peritonsillar abscess represent a new application of the well-established biomarker calprotectin. Hence, ascertainment of S100A8/A9 levels in the serum and saliva and application of the new PTA score is a useful diagnostic tool to differentiate between peritonsillar abscess, peritonsillar cellulitis, or acute tonsillitis during urgent outpatient consultation.

## Figures and Tables

**Table 1:** PTA score: Addition of one point each for symptoms like halitosis, trismus, uvula edema, unilateral swelling of the arched palate, and S100A8/A9 levels in serum and saliva higher than the cut-off values results in a score with a range from 0 to 6. High *S*_PTA_ values are associated with an increased probability of PTA.

Symptoms	Points
Halitosis	1
Trismus	1
Uvula edema	1
Unilateral swelling of the arched palate	1
Serum S100A8/A9 > 2550 ng/ml	1
Saliva S100A8/A9 > 8180 ng/ml	1

0–2: acute tonsillitis/peritonsillitis; ≥3: peritonsillar abscess.

**Table 2:** Probability of PTA in dependence on the *S*_PTA_ values. The likelihood for PTA increases with higher PTA score values. For *S*_PTA_ values ≥ 3, the overall probability of PTA is about 89%.

PTA score value	Probability of PTA	*n*
0	0%	21
1	0%	16
2	18%	17
3	92%	12
≥4	88%	25
